# Triglyceride–glucose index, neutrophil-to-lymphocyte ratio, and high-sensitivity C-reactive protein across albuminuria stages in type 2 diabetes

**DOI:** 10.1038/s41598-026-65484-7

**Published:** 2026-08-11

**Authors:** Marwa Mohamed Seyam, Hebatuallah Abdelhaleem Elhabiby, Zeinab Shafeek Hamed, Eman Rizk El Fiky, Manar Mohammed Elgebaly, Reham Abdelmageed Aboeida

**Affiliations:** 1 Internal Medicine Department, Faculty of Medicine, Tanta University, Tanta, 31511 Elgharbia Egypt; 2 Internal Medicine Department, Faculty of Medicine, Kafr El Sheikh University, Kafr El Sheikh, Egypt; 3 Clinical Pathology Department, Faculty of Medicine, Tanta University, Tanta, Egypt

**Keywords:** Diabetic kidney disease, Type 2 diabetes mellitus, Triglyceride–glucose index, Neutrophil–lymphocyte ratio, High-sensitivity C-reactive protein, Biomarkers, Diseases, Endocrinology, Medical research, Nephrology

## Abstract

Diabetic kidney disease (DKD) is a major microvascular complication of type 2 diabetes mellitus (T2DM) and a leading cause of progressive renal failure worldwide. Although urinary albumin-to-creatinine ratio (UACR) and estimated glomerular filtration rate remain the cornerstone measures for DKD assessment, simple and readily available biomarkers may provide complementary clinical information. In this cross-sectional study, 300 patients with T2DM were categorized into three groups according to UACR. The study evaluated and compared the triglyceride–glucose (TyG) index, neutrophil-to-lymphocyte ratio (NLR), and high-sensitivity C-reactive protein (hs-CRP) across albuminuria categories. All three biomarkers differed significantly among the albuminuria categories, with the highest levels observed in patients with macroalbuminuria. Receiver operating characteristic analyses demonstrated good discriminatory performance for albuminuria, with both NLR and hs-CRP exhibiting significantly greater discriminatory ability than the TyG index in several comparisons. In adjusted multinomial logistic regression analysis, NLR was independently associated with both microalbuminuria and macroalbuminuria, whereas the TyG index and hs-CRP were independently associated only with macroalbuminuria. These findings support the complementary role of these readily available biomarkers in the assessment of albuminuria in T2DM and provide a basis for future longitudinal studies to clarify their clinical significance.

## Introduction

Diabetes mellitus (DM) is a prevalent metabolic disorder characterized by prolonged hyperglycemia and chronic low-grade inflammation, both of which contribute to the development of microvascular complications, particularly diabetic kidney disease (DKD). DKD represents one of the long-term consequences of diabetes and affects a substantial proportion of patients, with approximately 20–50% are expected to develop some degree of renal involvement during the course of the disease. It is characterized by a progressive increase in urinary albumin excretion and a gradual decline in renal function, ultimately leading to end-stage kidney disease (ESKD)^1^.

Early detection and timely intervention may delay disease progression and improve renal outcomes; however, fewer than 20% of affected patients are diagnosed sufficiently early to receive appropriate management^2^.

Early detection of DKD is essential for slowing disease progression and improving renal outcomes. Current screening approaches predominantly rely on urinary albumin excretion and renal biopsy. Although microalbuminuria has long been employed as an early marker of DKD, it does not fully reflect the heterogeneity and complexity of the underlying disease processes. Renal biopsy, while providing definitive diagnostic information, is invasive, costly, and associated with procedural risks, which restricts its routine application. Consequently, dependence on these conventional methods may delay diagnosis and result in missed opportunities for timely intervention. Therefore, there is a need for simple, cost-effective, and non-invasive biomarkers that may provide complementary information regarding the metabolic and inflammatory alterations associated with DKD^3–5^.

Insulin resistance plays a central role in the pathogenesis of DKD. The triglyceride–glucose (TyG) index, calculated from fasting triglyceride and serum glucose levels, is a widely used surrogate marker of reduced insulin sensitivity that can be easily obtained in routine clinical practice. Emerging evidence suggests that elevated TyG index values are associated with an increased risk of DKD and other diabetes-related microvascular complications^6,7^.

Although the hyperinsulinemic-euglycemic clamp technique remains the gold standard for evaluating impaired insulin sensitivity, its complexity and limited practicality restrict its application in research settings. Similarly, indices such as the homeostatic model assessment of insulin resistance (HOMA-IR) rely on endogenous insulin measurements, which are unreliable in patients receiving exogenous insulin therapy and are not routinely performed in clinical practice^8^.

Consequently, the TyG index has emerged as a reliable and readily accessible alternative marker of reduced insulin sensitivity in patients with diabetes mellitus^9,10^.

Chronic systemic inflammation is recognized as a key factor in the development and progression of DKD. The neutrophil-to-lymphocyte ratio (NLR), derived from standard complete blood count parameters, has emerged as a readily available marker of systemic inflammation. Elevated NLR values have been linked to various inflammatory and metabolic disorders, and recent evidence indicates that increased NLR may be associated with the onset of renal injury in patients with diabetes mellitus^11–13^.

High-sensitivity C-reactive protein (hs-CRP) is a well-established systemic inflammatory marker synthesized predominantly by hepatocytes^14^. Insulin resistance is associated with increased hs-CRP concentrations, which subsequently triggers inflammatory cascades, enhances oxidative stress, and induces endothelial dysfunction. These pathological processes contribute to increased glomerular permeability, albuminuria, and progressive renal impairment in patients with diabetes mellitus^15,16^.

## Results

There were no significant differences in age or sex distribution among the three groups. Body mass index (BMI), diabetes duration, systolic and diastolic blood pressures, fasting serum glucose (FSG), glycated hemoglobin (HbA1c), total cholesterol, triglycerides, serum creatinine, and urinary albumin-to-creatinine ratio (UACR) increased significantly across albuminuria categories, whereas estimated glomerular filtration rate (eGFR) showed a significant decline. In addition, the triglyceride–glucose (TyG) index, neutrophil-to-lymphocyte ratio (NLR), and high-sensitivity C-reactive protein (hs-CRP) differed significantly across albuminuria categories, with the highest values observed in the macroalbuminuria group (Table 1).

**Table 1:** Comparison of demographic characteristics and clinical parameters among the study groups.

Variables	Group I (Normoalbuminuria) (n = 100)	Group II (Microalbuminuria) (n = 100)	Group III (Macroalbuminuria) (n = 100)	*P* value
Sex (M/F), n (%)	43 (43%)/57 (57%)	47 (47%)/53 (53%)	48 (48%)/52 (52%)	0.754 ^d^
Age (year)	50.50 (25.00)	50.50 (22.50)	52.00 (24.25)	0.251 ^e^
DM duration (year)	4.00 (6.00) ^a^	10.00 (4.00) ^b^	12.00 (6.00) ^c^	< 0.001 ^e^
BMI (kg/m^2^)	27.00 (2.14) ^a^	32.65 (2.40) ^b^	36.05 (1.30) ^c^	< 0.001 ^e^
SBP (mmHg)	120.00 (20.00) ^a^	145.00 (10.00) ^b^	160.00 (20.00) ^c^	< 0.001 ^e^
DBP (mmHg)	80.00 (10.00) ^a^	90.00 (0.00) ^b, f^	90.00 (10.00) ^c^	< 0.001^e^
FSG (mg/dL)	120.65 (21.45) ^a^	193.50 (28.85) ^b^	293.00 (20.00) ^c^	< 0.001 ^e^
HbA1c (%)	8.35 (1.25) ^a^	9.25 (0.80) ^b^	11.20 (1.50) ^c^	< 0.001 ^e^
TC (mg/dL)	198.30 (23.35) ^a^	248.00 (9.20) ^b^	287.50 (18.25) ^c^	< 0.001^e^
TG (mg/dL)	145.00 (18.62) ^a^	222.50 (38.83) ^b^	325.00 (43.38) ^c^	< 0.001 ^e^
SCr (mg/dL)	1.09 (0.23) ^a^	2.80 (0.42) ^b^	3.71 (2.72) ^c^	< 0.001 ^e^
UACR (mg/g)	20.10 (5.35) ^a^	153.35 (48.87) ^b^	630.70 (210.65) ^c^	< 0.001 ^e^
eGFR (mL/min/1.73 m^2^)	69.59 (22.82) ^a^	22.79 (5.73) ^b^	17.06 (51.63) ^c^	< 0.001 ^e^
TyG index	9.20 (1.80) ^a^	9.90 (0.20) ^b^	10.70 (0.20) ^c^	< 0.001 ^e^
NLR	1.91 (0.44) ^a^	3.37 (0.52) ^b^	5.00 (0.79) ^c^	< 0.001 ^e^
Hs-CRP (mg/L)	0.96 (0.44) ^a^	1.97 (0.83) ^b^	4.33 (1.61) ^c^	< 0.001 ^e^

*P* values are two-tailed. Continuous variables are presented as median (IQR), and categorical variables as n (%). Overall comparisons were performed using the Kruskal–Wallis test, followed by pairwise comparisons using the Mann–Whitney U test with Bonferroni correction. For pairwise comparisons, statistical significance was defined as adjusted *P* < 0.0167. Superscript letters indicate significant pairwise differences:

^a ^Normoalbuminuria versus microalbuminuria.

^b ^Normoalbuminuria versus macroalbuminuria.

^c ^Microalbuminuria versus macroalbuminuria.

^d ^Chi-square test.

^e ^Kruskal–Wallis test.

^f ^The zero IQR observed for DBP in Group II reflects clustering of recorded values around the median and does not indicate a data entry or calculation error. No similar clustering affected the interpretation of other continuous variables.

In the correlation analysis, the triglyceride–glucose (TyG) index, neutrophil-to-lymphocyte ratio (NLR), and high-sensitivity C-reactive protein (hs-CRP) demonstrated significant positive correlations with all the studied parameters, except for estimated glomerular filtration rate (eGFR), which exhibited significant negative correlations (Table 2).

**Table 2:** Correlation of TyG Index, NLR and hs-CRP with the different studied parameters.

Variables	TyG index		NLR		HS-CRP (mg/L)	
	rs ^a^	*p* value	rs ^a^	*p* value	rs ^a^	*p* value
DM duration (year)	0.268	< 0.001	0.448	< 0.001	0.508	< 0.001
BMI (kg/m^2^)	0.443	< 0.001	0.566	< 0.001	0.659	< 0.001
SBP (mmHg)	0.361	< 0.001	0.583	< 0.001	0.627	< 0.001
DBP (mmHg)	0.274	< 0.001	0.536	< 0.001	0.548	< 0.001
FSG (mg/dL)	0.416	< 0.001	0.646	< 0.001	0.626	< 0.001
HbA1c (%)	0.256	< 0.001	0.345	< 0.001	0.309	< 0.001
TC (mg/dL)	0.356	< 0.001	0.594	< 0.001	0.638	< 0.001
TG (mg/dL)	0.411	< 0.001	0.588	< 0.001	0.664	< 0.001
SCr (mg/dL)	0.291	< 0.001	0.417	< 0.001	0.443	< 0.001
UACR (mg/g)	0.431	< 0.001	0.646	< 0.001	0.694	< 0.001
eGFR (mL/min/1.73 m^2^)	− 0.287	< 0.001	− 0.413	< 0.001	− 0.434	< 0.001
NLR	0.343	< 0.001	–	–	0.597	< 0.001
Hs-CRP (mg/L)	0.409	< 0.001	0.597	< 0.001	–	–
TyG index	–	–	0.343	< 0.001	0.409	< 0.001

Two-tailed *P* values are shown. α = 0.05; *P* < 0.05 considered significant.

^a ^Spearman correlation.

**Fig. 1:**
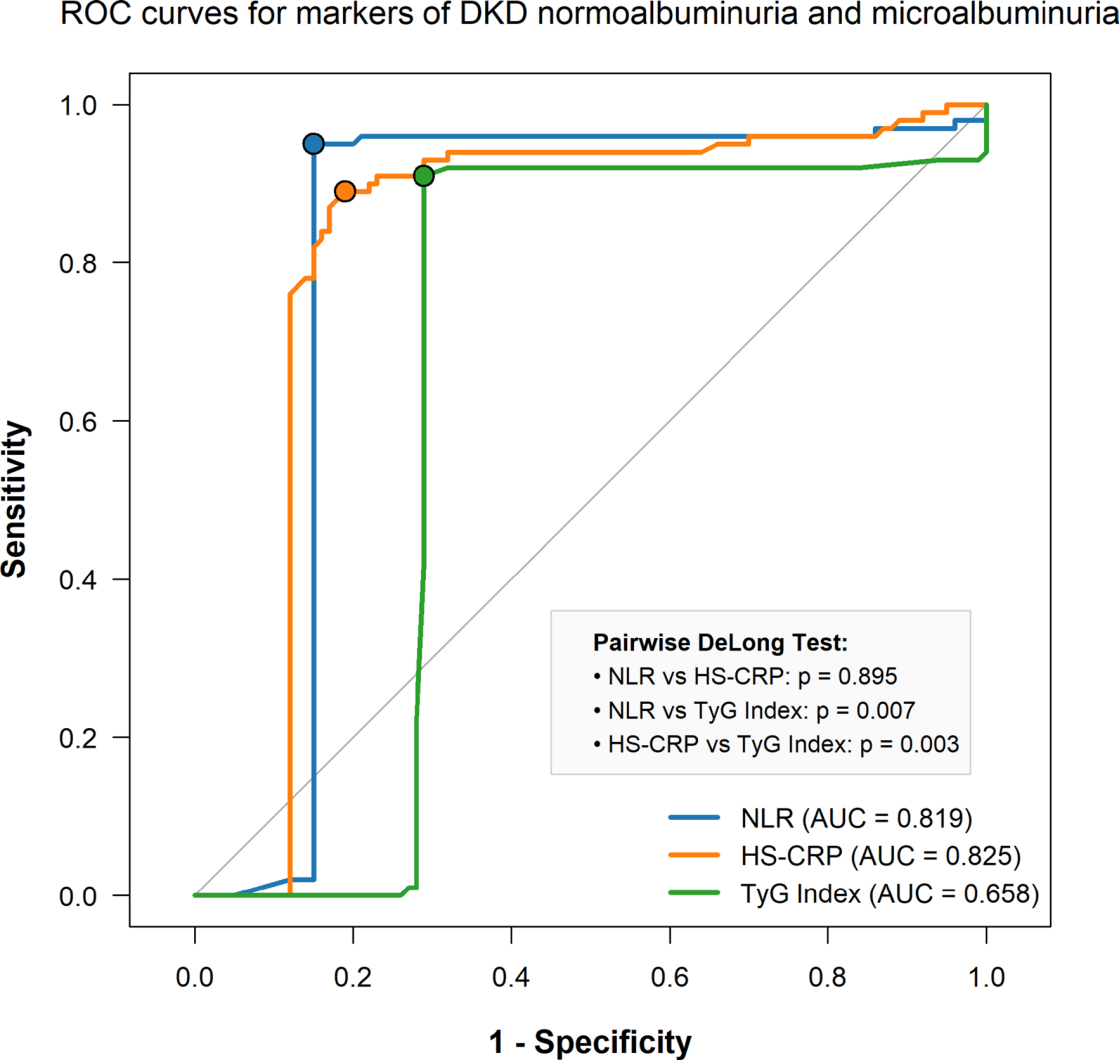
Receiver operating characteristic (ROC) curves for TyG index, NLR, and hs-CRP in discriminating between patients with normoalbuminuria and microalbuminuria. Curves represent TyG index (green), NLR (blue), and hs-CRP (orange). Optimal cut-off points are indicated by circles, and pairwise comparisons of the area under the curve (AUC) using DeLong’s test are provided on the figure.

Receiver operating characteristic (ROC) analysis was performed to evaluate the discriminatory ability of the investigated biomarkers between predefined albuminuria categories. For differentiating normoalbuminuria (Group I) from microalbuminuria (Group II), hs-CRP demonstrated the highest discriminatory performance (AUC = 0.825), followed closely by NLR (AUC = 0.819), whereas the TyG index showed the lowest discriminative performance (AUC = 0.658). Pairwise comparisons of the ROC curves using the DeLong test demonstrated that both NLR and hs-CRP had significantly higher AUCs than the TyG index (*p* = 0.007 and *p* = 0.003, respectively), whereas the difference between NLR and hs-CRP was not statistically significant (*p* = 0.895). The optimal cut-off values and the corresponding sensitivity, specificity, positive predictive value, and negative predictive value are presented in Table 3 and Fig. 1.

**Table 3:** Discriminatory performance of the TyG index, NLR, and hs-CRP for differentiating normoalbuminuria from microalbuminuria.

Biomarker	AUC ^a^	95% CI ^b^	Youden Index	Optimal cut-off	Sensitivity (%)	Specificity (%)	PPV ^c^ (%)	NPV ^d^ (%)
TyG index	0.658	0.570–0.746	0.62	9.5	91	71	76	89
NLR	0.819	0.742–0.889	0.80	2.5	95	85	86	94
Hs-CRP (mg/L)	0.825	0.754–0.888	0.70	1.34	87	83	84	87

^a ^Area Under the Curve.

^b ^Confidence interval.

^c ^Positive predictive value.

^d ^Negative predictive value.

**Fig. 2:**
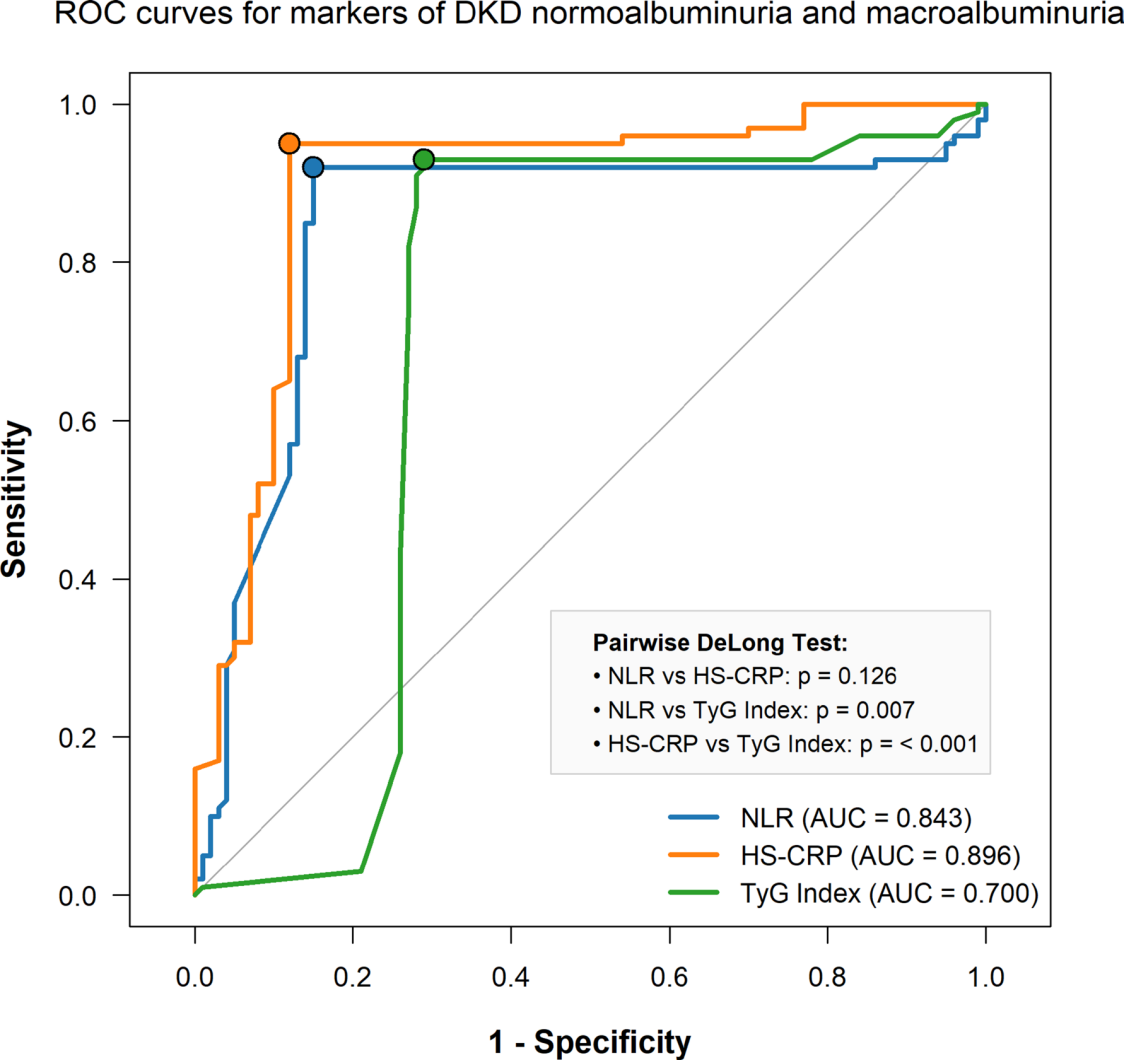
Receiver operating characteristic (ROC) curves for TyG index, NLR, and hs-CRP in discriminating between patients with normoalbuminuria and macroalbuminuria. Curves represent TyG index (green), NLR (blue), and hs-CRP (orange). Optimal cut-off points are indicated by circles, and pairwise comparisons of the area under the curve (AUC) using DeLong’s test are provided on the figure.

Receiver operating characteristic (ROC) analysis was performed to evaluate the discriminatory ability of the investigated biomarkers between normoalbuminuria (Group I) and macroalbuminuria (Group III). Among the studied biomarkers, hs-CRP demonstrated the highest discriminatory performance (AUC = 0.895), followed by NLR (AUC = 0.843), whereas the TyG index showed the lowest discriminative performance (AUC = 0.700). Pairwise comparisons of the ROC curves using the DeLong test demonstrated that both NLR and hs-CRP had significantly higher AUCs than the TyG index (*p* = 0.007 and p < 0.001, respectively), whereas the difference between NLR and hs-CRP was not statistically significant (*p* = 0.126). The optimal cut-off values and the corresponding sensitivity, specificity, positive predictive value, and negative predictive value are presented in Table 4 and Fig. 2.

**Table 4:** Discriminatory performance of the TyG index, NLR, and hs-CRP for differentiating normoalbuminuria from macroalbuminuria.

Biomarker	AUC ^a^	95% CI ^b^	Youden Index	Optimal cut-off	Sensitivity (%)	Specificity (%)	PPV ^c^ (%)	NPV ^d^ (%)
TyG index	0.700	0.613–0.780	0.64	9.9	93	71	76	91
NLR	0.843	0.779–0.904	0.77	4	92	85	86	91
Hs-CRP (mg/L)	0.89**6**	0.844–0.941	0.83	1.98	95	88	89	95

^a ^Area Under the Curve.

^b ^Confidence interval.

^c ^Positive predictive value.

^d ^Negative predictive value.

**Fig. 3:**
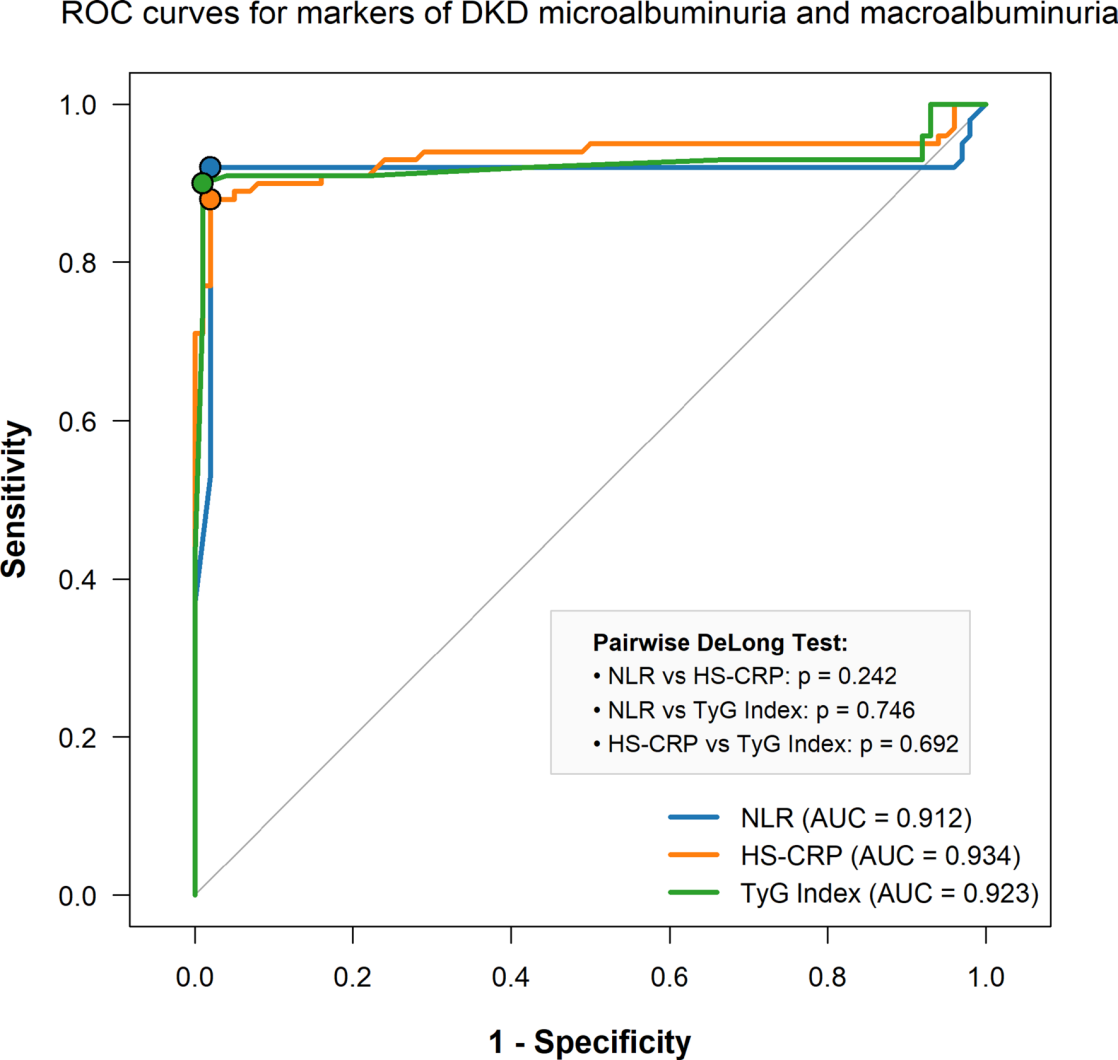
Receiver operating characteristic (ROC) curves for TyG index, NLR, and hs-CRP in discriminating between patients with microalbuminuria and macroalbuminuria. Curves represent TyG index (green), NLR (blue), and hs-CRP (orange). Optimal cut-off points are indicated by circles, and pairwise comparisons of the area under the curve (AUC) using DeLong’s test are provided on the figure.

Receiver operating characteristic (ROC) analysis was performed to evaluate the discriminatory ability of the investigated biomarkers between microalbuminuria (Group II) and macroalbuminuria (Group III). All three biomarkers demonstrated excellent diagnostic performance, with AUC values exceeding 0.90. hs-CRP showed the highest discriminatory performance (AUC = 0.934), followed by the TyG index (AUC = 0.923) and NLR (AUC = 0.912). Pairwise comparisons of the ROC curves using the DeLong test revealed no statistically significant differences among the three biomarkers (NLR vs. hs-CRP, *p* = 0.242; NLR vs. TyG index, *p* = 0.746; hs-CRP vs. TyG index, *p* = 0.692). The optimal cut-off values and the corresponding sensitivity, specificity, positive predictive value, and negative predictive value are presented in Table 5 and Fig. 3.

**Table 5:** Discriminatory performance of the TyG index, NLR, and hs-CRP for differentiating microalbuminuria from macroalbuminuria.

Biomarker	AUC ^a^	95% CI ^b^	Youden Index	Optimal cut-off	Sensitivity (%)	Specificity (%)	PPV ^c^ (%)	NPV ^d^ (%)
TyG index	0.923	0.868–0.967	0.89	10.3	90	99	99	91
NLR	0.912	0.852–0.960	0.90	4	92	98	98	93
Hs-CRP (mg/L)	0.934	0.888–0.971	0.86	3.02	88	98	98	89

^a ^Area Under the Curve.

^b ^Confidence interval.

^c ^Positive predictive value.

^d ^Negative predictive value.

Univariate odds ratios are presented separately, whereas adjusted odds ratios were obtained from the multinomial logistic regression model after simultaneous adjustment for all covariates. The overall model showed good fit (McFadden’s pseudo-R^2^ = 0.806; likelihood ratio χ^2^ = 531, df = 16, P < 0.001), with no significant multicollinearity detected (VIF range, 1.06–2.34). The multinomial regression model included nine parameters and yielded an events-per-variable ratio of approximately 11.1. Diabetes duration, lower eGFR, and NLR were independently associated with both microalbuminuria and macroalbuminuria. TyG index and hs-CRP showed significant associations predominantly in the macroalbuminuria versus normoalbuminuria comparison (Group III vs. Group I) in the primary multinomial regression model (Table 6). For TyG index, the estimated OR was 9.28 (95% CI 1.96–43.95; *p* = 0.005). However, the wide confidence interval indicated limited precision of the estimate. A sensitivity analysis using Firth’s penalized logistic regression yielded a more conservative estimate (OR = 2.02, 95% CI 0.79–5.52; *p* = 0.140), suggesting that the strength of the observed association should be interpreted cautiously.

**Table 6:** Univariate logistic regression and adjusted multinomial logistic regression analyses of albuminuria categories using normoalbuminuria (Group I) as the reference category.

Variables	Univariate logistic regression		Adjusted multinomial logistic regression			
	OR ^a^ (95% CI ^b^)	*p* value	Group II versus Group I		Group III versus Group I	
			OR ^a^ (95% CI ^b^)	*p* value	OR ^a^ (95% CI ^b^)	*p* value
Age ( year )	1.01 (0.99–1.03)	0.151	0.89 (0.80–0.98)	0.022	0.89 (0.80–0.99)	0.031
Sex	1.20 (0.74–1.94)	0.461	0.27 (0.03–1.98)	0.201	0.16 (0.01–1.36)	0.094
DM duration ( year )	1.66 (1.48–1.86)	< 0.001	2.03 (1.41–2.91)	< 0.001	2.16 (1.50–3.12)	< 0.001
BMI (kg/m^2^)	2.17(1.84–2.57)	< 0.001	1.64 (1.22–2.21)	< 0.001	2.29 (1.65–3.16)	< 0.001
HbA1c (%)	1.19 (1.07–1.34)	0.002	0.91 (0.55–1.51)	0.736	1.02 (0.59–1.75)	0.939
eGFR (mL/min/1.73 m^2^)	0.84 (0.80–0.88)	< 0.001	0.89 (0.85–0.94)	< 0.001	0.94 (0.90–0.98)	0.013
TyG index	2.73 (1.94–3.86)	< 0.001	0.83 (0.21–3.18)	0.787	9.28 (1.96–43.95)	0.005
NLR	3.13 (2.39–4.08)	< 0.001	3.40 (1.53–7.55)	0.002	4.69 (1.94–11.30)	< 0.001
Hs-CRP (mg/L)	2.72 (2.08–3.57)	< 0.001	1.00 (0.48–2.11)	0.985	3.82 (1.76–8.26)	< 0.001

Two-tailed *P* values are shown. α = 0.05; *P* < 0.05 considered significant.

^a^ Odds ratio.

^b^ confidence interval.

Univariate odds ratios were obtained from separate unadjusted logistic regression models for each parameter. Adjusted odds ratios were derived from the multinomial logistic regression model including age, sex, diabetes duration, BMI, HbA1c, eGFR, TyG index, NLR, and hs-CRP simultaneously.

## Discussion

While UACR and eGFR remain the cornerstone measures of DKD assessment, the identification of readily available biomarkers reflecting metabolic and inflammatory disturbances may provide complementary clinical information. However, they should be considered adjunctive rather than replacement markers.

The triglyceride–glucose (TyG) index, neutrophil-to-lymphocyte ratio (NLR), and high-sensitivity C-reactive protein (hs-CRP) were significantly higher across albuminuria categories, with the highest values observed in the macroalbuminuria group. These findings are consistent with previous studies^17–19^.

Our results revealed strong positive correlations between the triglyceride–glucose (TyG) index, neutrophil-to-lymphocyte ratio (NLR), and high-sensitivity C-reactive protein (hs-CRP) and other parameters, including diabetes duration, body mass index (BMI), systolic and diastolic blood pressure, fasting serum glucose (FSG), glycated hemoglobin (HbA1c), total cholesterol (TC), triglycerides (TG), serum creatinine, urinary albumin-to-creatinine ratio (UACR), along with negative correlations with estimated glomerular filtration rate (eGFR). These findings are partially supported by previous studies^18–21^.

These findings support an association between inflammatory activity, insulin resistance, poor glycemic control, dyslipidemia, hypertension, and impaired renal function in patients with type 2 diabetes mellitus (T2DM).

Recent evidence indicates that diabetic kidney disease is a multifactorial disorder driven by complex interactions among metabolic dysregulation, chronic inflammation, tissue hypoxia, and progressive renal fibrosis. Prospective real-world cohort studies have further emphasized that reliable identification of prognostic factors for DKD progression requires longitudinal follow-up rather than cross-sectional assessment. In addition, chronic renal hypoxia, a hallmark of DKD, promotes hypoxia-inducible factor-1 alpha (HIF-1α) activation, which induces phenotypic changes in tubular epithelial cells and stimulates profibrotic pathways through epithelial-mesenchymal transition (EMT)-related transcription factors. These processes contribute to tubulointerstitial fibrosis and progressive deterioration of renal structure and function^22^. Furthermore, podocyte injury disrupts the integrity of the glomerular filtration barrier, leading to progressive albuminuria^23^, while hyperglycemia-induced macrophage recruitment and activation sustain chronic inflammation through the release of pro-inflammatory and profibrotic mediators, thereby accelerating renal fibrosis and proteinuria^24,25^.

Within this biological framework, the higher levels of TyG index, NLR, and hs-CRP observed across the different albuminuria categories are biologically consistent with the metabolic and inflammatory disturbances accompanying DKD.

Although these biomarkers do not directly assess hypoxia-induced metabolic reprogramming, macrophage activation, EMT, or renal fibrosis, their associations with albuminuria may indirectly reflect the cumulative effects of these pathogenic processes. Nevertheless, because of the cross-sectional design, these findings should be interpreted as associations rather than evidence of causality or prognostic utility, and future prospective longitudinal studies are required to determine whether these biomarkers provide additional prognostic information beyond conventional renal markers such as UACR and eGFR and whether they improve risk stratification in clinical practice. ROC analyses were performed to evaluate the discriminatory performance of the investigated biomarkers across predefined albuminuria categories. However, because these categories represent clinically distinct groups with differences in metabolic status, inflammatory activity, and renal function, the observed discrimination may partly reflect underlying between-group heterogeneity. Therefore, these findings should be interpreted as measures of differentiation between existing albuminuria categories rather than as evidence of independent diagnostic or predictive utility. DeLong pairwise comparisons demonstrated significantly higher AUCs for NLR and hs-CRP than for the TyG index in comparisons involving normoalbuminuria, whereas no statistically significant differences were observed among the three biomarkers in distinguishing microalbuminuria from macroalbuminuria. These findings suggest that inflammatory markers may have a closer association with different albuminuria categories than the metabolic insulin resistance surrogate reflected by the TyG index. In the adjusted multinomial logistic regression model, diabetes duration, BMI, lower eGFR, and NLR remained independently associated with both microalbuminuria and macroalbuminuria. In contrast, TyG index and hs-CRP showed independent associations predominantly in the macroalbuminuria comparison. Although the direction of the association between TyG index and macroalbuminuria remained consistent in the primary analysis, the wide confidence interval indicates uncertainty in the precision of the estimate. Furthermore, sensitivity analysis using Firth’s penalized regression resulted in a more conservative and non-significant estimate, suggesting that the magnitude of the observed association may be influenced by sparse-data effects and should therefore be interpreted cautiously. Within the cross-sectional framework of the present study, TyG may be associated with more advanced albuminuria; however, these findings should not be interpreted as evidence of temporal progression or causality.

The absence of independent associations between TyG index and hs-CRP with microalbuminuria after multivariable adjustment suggests that these biomarkers did not demonstrate additional independent associations beyond the clinical and renal variables included in the model for early albuminuria stages. Their associations were mainly observed in macroalbuminuria, suggesting a possible relationship with the metabolic and inflammatory alterations accompanying more advanced albuminuria. However, these findings do not support the use of TyG index or hs-CRP as standalone biomarkers for early DKD detection.

Several limitations should be considered when interpreting the findings of the present study. First, the cross-sectional design precludes causal inference and does not allow assessment of temporal relationships or predictive associations between the investigated biomarkers and diabetic kidney disease progression.

Second, although patients were categorized into clinically relevant albuminuria groups according to KDIGO criteria, these groups inherently differed in several baseline characteristics, including metabolic status, inflammatory activity, blood pressure, and renal function. These differences represent the clinical spectrum of albuminuria severity in diabetic kidney disease but may also introduce residual confounding and influence the magnitude of observed associations and discriminatory performance. Although multivariable adjustment was performed to account for important covariates, residual confounding related to disease severity cannot be completely excluded.

Third, sample size estimation was performed during the study planning stage using a parametric approach (one-way ANOVA), whereas the final analyses relied primarily on non-parametric methods because several variables were not normally distributed. Although this represents a common approach in observational studies, the assumptions underlying the sample size estimation did not fully correspond to the final analytical strategy and should therefore be acknowledged as a methodological limitation.

Fourth, although each albuminuria group included 100 participants, the adjusted effect estimate for the TyG index in the macroalbuminuria comparison showed limited precision, as reflected by the wide confidence interval. Furthermore, the Firth penalized regression sensitivity analysis resulted in a more conservative estimate, suggesting that the magnitude of the observed association may be sensitive to model specification.

Fifth, although systolic blood pressure differed significantly across albuminuria categories and showed correlations with the investigated biomarkers, it was not included in the final multivariable model. When SBP was incorporated, it resulted in substantial inflation of regression coefficients and odds ratios, suggesting instability of parameter estimates in the current dataset. Therefore, the final model was retained without SBP to avoid potentially unreliable estimates. Nevertheless, residual confounding related to blood pressure cannot be completely excluded.

Sixth, the present study evaluated the individual associations and discriminatory performance of TyG index, NLR, and hs-CRP separately. Although a combined biomarker model may potentially provide improved discrimination, development and validation of such an integrated model were beyond the scope of this cross-sectional study. Future studies with larger sample sizes, prospective follow-up, and external validation are needed to determine whether combined metabolic and inflammatory biomarker models provide additional clinical value beyond conventional renal markers such as UACR and eGFR.

Seventh, although medications known to substantially influence inflammatory biomarkers and metabolic parameters, including statins, sodium-glucose cotransporter 2 (SGLT2) inhibitors, and corticosteroids, were excluded to minimize treatment-related confounding, residual confounding related to antihypertensive therapy, other concomitant medications, diabetes duration, and disease chronicity may still have influenced the observed associations. Furthermore, excluding patients receiving commonly used therapies such as statins and SGLT2 inhibitors may limit the generalizability of the findings to broader real-world populations. Because medication exposure among excluded individuals was not systematically recorded, the proportion of patients who would meet these exclusion criteria in a typical diabetes clinic population could not be determined.

Finally, this was a single-center study that included patients with established diabetic kidney disease and persistent albuminuria. Therefore, the findings may not be fully generalizable to individuals with type 2 diabetes without established DKD, patients with early-stage kidney disease, screening populations, treatment-naïve populations, or other clinical settings. External validation in larger multicenter cohorts is warranted before broader clinical application.

In conclusion, the triglyceride–glucose (TyG) index, neutrophil-to-lymphocyte ratio (NLR), and high-sensitivity C-reactive protein (hs-CRP) were associated with different albuminuria categories among patients with type 2 diabetes mellitus (T2DM). NLR demonstrated independent associations with both microalbuminuria and macroalbuminuria, whereas TyG index and hs-CRP showed associations predominantly with macroalbuminuria in the primary adjusted model. However, the association between TyG index and macroalbuminuria was attenuated in sensitivity analyses using Firth’s penalized regression, indicating that this finding should be interpreted cautiously.

Although hs-CRP and NLR demonstrated higher discriminatory performance than the TyG index in most ROC comparisons, these findings should be interpreted as differences between predefined albuminuria categories rather than evidence of diagnostic, predictive, or prognostic utility. Overall, these findings suggest that inflammatory and metabolic biomarkers are associated with albuminuria categories in T2DM. However, their incremental clinical value beyond established renal markers requires confirmation in prospective studies.

## Materials and methods

This cross-sectional study included 300 patients with type 2 diabetes mellitus who were recruited from the Endocrinology Unit, Internal Medicine Department, Tanta University Hospital, from August 2025 to December 2025. The study protocol was approved by the Medical Ethical Committee of the Faculty of Medicine, Tanta University, approval number (36264PR1318/8/25). As this was a cross-sectional observational study without any interventional procedures, clinical trial registration was not applicable. All procedures involving human participants were performed in accordance with the ethical standards of the institutional and/or national research committee and with the 1964 Helsinki Declaration and its later amendments.

Written informed consent was obtained from all patients included in the study.

The study enrolled patients with T2DM who fulfilled the World Health Organization (WHO) diagnostic criteria. Patients were categorized into three groups based on their urinary albumin-to-creatinine ratio (UACR). Group I included 100 diabetic patients with normoalbuminuria (UACR < 30 mg/g), Group II comprised 100 diabetic patients with microalbuminuria (UACR 30–300 mg/g), and Group III consisted of 100 diabetic patients with macroalbuminuria (UACR > 300 mg/g). These classifications were based on the KDIGO 2024 clinical practice guideline for the evaluation and management of chronic kidney disease^26^. All patients with albuminuria included in the study had a prior diagnosis of DKD before enrollment, and albuminuria was confirmed by repeat UACR testing over a period of at least 3 months, as required by KDIGO criteria.

Patients with severe cardiac, hepatic, renal, or other organ dysfunction; tumors; type 1 diabetes or other specific types of diabetes; gestational diabetes; acute complications such as diabetic ketoacidosis or hyperglycemic hyperosmolar state; conditions affecting high-sensitivity C-reactive protein (hs-CRP) and neutrophil-to-lymphocyte ratio (NLR), including acute infections, chronic inflammatory disorders, anemia, smoking, or use of medications such as corticosteroids, statins, and sodium-glucose co-transporter 2 inhibitors (SGLT2); and conditions impacting urinary protein excretion, such as nephrotic or nephritic syndrome, urolithiasis, urinary tract infection, and other acute infections, were excluded from the study. Patients receiving corticosteroids, statins, or SGLT2 inhibitors were excluded because these medications may substantially influence systemic inflammatory markers, insulin resistance, and metabolic parameters, thereby potentially confounding the associations between the investigated biomarkers and albuminuria. All participants underwent comprehensive history taking, thorough clinical examination including blood pressure measurements, body mass index (BMI) calculation, and laboratory investigations, including complete blood count, fasting serum glucose (FSG), glycated hemoglobin (HbA1c), total cholesterol (TC), triglycerides (TG), serum creatinine (SCr), estimated glomerular filtration rate (eGFR), urinary albumin-to-creatinine ratio (UACR), and high-sensitivity C-reactive protein (hs-CRP).

Blood pressure measurements were obtained using a validated automated sphygmomanometer under standardized conditions. Measurements were performed in a seated position after at least 5 min of rest in a quiet environment. An appropriately sized cuff was applied to the non-dominant arm at heart level. Two consecutive readings were recorded at 1–2 min intervals, and the mean of the two readings was used for analysis. A third measurement was obtained if a difference greater than 5 mmHg was observed between the first two readings.

Calculations:

BMI was calculated as weight (kg) divided by height squared (m^2^).

Estimated glomerular filtration rate (eGFR) was calculated using the 2021 Chronic Kidney Disease Epidemiology Collaboration (CKD-EPI) creatinine equation, without application of a race coefficient^26^.

The TyG index was calculated using the following formula:

$${\mathrm{TyG}}\;{\mathrm{index}} = {\text{ln }}[{\text{TG }}\left( {\mathrm{mg/dL}} \right) \times {\text{FSG }}\left( {\mathrm{mg/dL}} \right){/2}].$$

The neutrophil-to-lymphocyte ratio (NLR) was determined as the ratio of the absolute neutrophil count to the absolute lymphocyte count obtained from a complete blood count.

Quality control procedures were implemented throughout the study. All demographic, clinical, and laboratory data were checked for completeness and consistency before analysis. Any ambiguous or inconsistent entries were cross-checked against the original data collection forms and laboratory reports to ensure data accuracy.

### Blood sampling

Two milliliters of fasting peripheral blood were collected in EDTA vacutainer tubes for complete blood count (CBC) and glycated hemoglobin (HbA1c) analysis using sterile disposable syringes under strict aseptic conditions. Additionally, three milliliters of blood were collected into sterile, plain tubes, allowed to clot, and the serum was separated for the measurement of fasting serum glucose, total cholesterol, triglycerides, hs-CRP, and serum creatinine.

Complete blood count (CBC) was obtained using an automated cell counter ERMA PCE-210N (Erma Inc., Tokyo, Japan). Glycated hemoglobin (HbA1c) was measured by high-performance liquid chromatography (HPLC) using the TOSOH G8 analyzer (Tosoh Bioscience, Tokyo, Japan). High-sensitivity C-reactive protein (hs-CRP) was quantified by nephelometry using the Atellica NEPH 630 system (Siemens Healthineers, Marburg, Germany). Serum triglycerides, total cholesterol, glucose, and creatinine were analyzed using the automated Konelab system (Thermo Fisher Scientific, Waltham, MA, USA). Urine samples were collected in sterile, dry cups for the assessment of microalbuminuria using immunoturbidimetric latex COD 31,924 (BioSystems S.A., Barcelona, Spain) and creatinine using the automated Konelab system (Thermo Fisher Scientific, Waltham, MA, USA) to calculate the urinary albumin-to-creatinine ratio (UACR).

### Sample size

Sample size estimation was performed during the study planning stage using the one-way ANOVA framework based on Cohen’s standardized effect size (*f*), which is a widely accepted approach for sample size estimation in multi-group studies with continuous outcomes. Standard deviations and distribution ranges were obtained from previous studies. Assuming a moderate-to-large effect size (Cohen’s *f* = 0.3203), a significance level (α) of 0.05, and 80% statistical power, the minimum required sample size was estimated to be 97 participants per group. This standardized approach provides a common effect size metric for continuous outcomes measured on different scales. To account for potential dropouts, the sample size was increased to 100 participants per group, resulting in a total sample of 300 participants. After data collection, the distribution of continuous variables was assessed using the Shapiro–Wilk test, and non-parametric methods were applied for variables that did not satisfy the normality assumption.

### Statistical analysis

Statistical analyses were performed using the Python programming language (v3.13), with Pandas and NumPy used for data management and preprocessing. Descriptive statistics, group comparisons, and diagnostic performance analyses were conducted using SciPy^27^ and Scikit-learn libraries^28^. Multinomial logistic regression modeling and model fit evaluation were performed using Jamovi (Version 2.7). The Shapiro–Wilk test was used to assess normality of continuous variables. Continuous variables were expressed as mean ± standard deviation for normally distributed data and as median (interquartile range, IQR) for non-normally distributed data.

### Comparative analyses

For comparisons across the three study groups, one-way ANOVA was applied for parametric variables, while the Kruskal–Wallis test was used for non-parametric variables. When overall significance was detected, post-hoc pairwise comparisons were performed using the Mann–Whitney U test. To control for Type I error inflation, Bonferroni correction was applied, with statistical significance set at *P* < 0.0167.

Associations between categorical variables were evaluated using the Chi-square test (χ^2^), while Fisher’s exact test was used when expected cell counts were less than 5.

### Correlation and diagnostic performance analyses

Spearman’s correlation coefficient (rs) was used to assess relationships between non-normally distributed variables.

Receiver operating characteristic (ROC) curve analysis was performed using binary classification to evaluate the discriminatory performance of the TyG index, NLR, and hs-CRP across clinically relevant albuminuria comparisons. ROC analyses were conducted for the following comparisons: Normoalbuminuria versus microalbuminuria, normoalbuminuria versus macroalbuminuria, and microalbuminuria versus macroalbuminuria. The area under the curve (AUC) was calculated to assess diagnostic performance. Optimal cut-off values were determined using the maximum Youden index (J = sensitivity + specificity − 1). The corresponding sensitivity, specificity, positive predictive value (PPV), and negative predictive value (NPV) were calculated at the optimal threshold. Pairwise comparisons of AUCs were performed using DeLong’s test, with statistical significance defined as P < 0.05. Multivariable regression analysis:

Because albuminuria categories represent clinically distinct states with expected differences in disease burden, multivariable multinomial logistic regression was performed to account for potential confounding factors.

Multinomial logistic regression analysis was performed to identify variables independently associated with albuminuria stage. Albuminuria category was used as the dependent variable, with normoalbuminuria (Group I) serving as the reference category. A single multinomial model incorporating all three albuminuria categories was fitted, yielding adjusted odds ratios (ORs) for microalbuminuria (Group II) and macroalbuminuria (Group III), each relative to Group I.

The final model included clinically relevant covariates selected based on biological plausibility and previous evidence, including age, sex, BMI, diabetes duration, HbA1c, eGFR, TyG index, NLR, and hs-CRP. Systolic blood pressure was evaluated as a potential covariate; however, its inclusion resulted in unstable parameter estimates with extreme odds ratios and wide confidence intervals, suggesting quasi-complete separation and reduced model interpretability. Therefore, SBP was not retained in the final model. Multicollinearity was assessed using the variance inflation factor (VIF). Model fit was evaluated using the likelihood ratio test and McFadden’s pseudo-R^2^. The events-per-variable (EPV) ratio was calculated to assess model complexity, and regression coefficients, standard errors, and Wald statistics were reviewed for parameter estimation. A sensitivity analysis using Firth’s penalized logistic regression was performed for the TyG index estimate in the macroalbuminuria comparison to assess the robustness of the regression findings.

### Statistical significance

All statistical tests were two-sided. A *P* value < 0.05 was considered statistically significant unless otherwise specified. Bonferroni-adjusted thresholds were applied for multiple pairwise comparisons.

## Data Availability

The datasets generated and analyzed during the current study are available from the corresponding author upon reasonable request.

## Abbreviations

DKD: Diabetic kidney disease
T2DM: Type 2 diabetes mellitus
UACR: Urinary albumin-to-creatinine ratio
TyG: Triglyceride–glucose
NLR: Neutrophil–lymphocyte ratio
Hs-CRP: High-sensitivity C-reactive protein
ROC: Receiver operating characteristic
DM: Diabetes mellitus
HOMA-IR: Homeostatic model assessment of insulin resistance
WHO: World Health Organization
BMI: Body mass index
FSG: Fasting serum glucose
HbA1c: Glycated hemoglobin
TC: Total cholesterol
TG: Triglyceride
SCr: Serum creatinine
eGFR: Estimated glomerular filtration rate
ESKD: End-stage kidney disease
CKD-EPI: Chronic kidney disease epidemiology collaboration
HIF-1α: Hypoxia-inducible factor-1 alpha
EMT: Epithelial-mesenchymal transition
